# Effects of a Prolonged Exclusive Human Milk-Based Diet on Structural and Functional Brain Maturation in Very Preterm Infants: An Ancillary Analysis of the NEOVASC Trial

**DOI:** 10.3390/nu18091321

**Published:** 2026-04-22

**Authors:** Wolfgang Mitterer, Christoph Hochmayr, Maria Waltner-Romen, Maria Sappler, Marlene Hammerl, Lena Gatterer, Vera Neubauer, Ursula Kiechl-Kohlendorfer

**Affiliations:** 1 Department of Pediatrics II, Neonatology, Medical University of Innsbruck, Anichstraße 35, A-6020 Innsbruck, Austria; 2 Research Centre on Vascular Ageing and Stroke, VASCage GmbH, 6020 Innsbruck, Austria

**Keywords:** preterm infants, human milk-based diet, human milk fortification, neonatal nutrition, brain maturation, neurodevelopment, magnetic resonance imaging, diffusion tensor imaging, general movements assessment, motor development

## Abstract

**Background/Objectives:** Early postnatal nutrition is a modifiable determinant of brain maturation in preterm infants. Exclusive human milk-based diets (EHMD) are associated with improved neurodevelopmental outcomes. The objective of this exploratory ancillary analysis of the NEOVASC randomized controlled trial was to determine whether prolonging an exclusive human milk-based diet, specifically through continued human milk-based fortification until 36 weeks postmenstrual age, is associated with differences in early brain structure and functional motor development compared with earlier introduction of bovine milk-based fortifier or formula at 32 weeks postmenstrual age. **Methods:** This ancillary study of the NEOVASC trial included preterm infants (<32 gestational weeks and birthweight of 500–1250 g) randomized to either prolonged EHMD until 36 weeks PMA or a diet introducing bovine milk-based fortifier or formula from 32 weeks. Quantitative brain metrics, fractional anisotropy (FA), and apparent diffusion coefficient (ADC) were analyzed at 40 weeks PMA. Functional maturation was assessed repetitively using the General Movement Optimality Score (GMOS) (34, 36, and 40 weeks PMA) and Motor Optimality Score (52 weeks PMA). **Results:** Fifty-four infants were included. Groups did not differ in brain growth metrics. After adjustment for imbalances in clinical characteristics, no FA or ADC differences remained statistically significant. GMOS at 40 weeks PMA was higher in the intervention group, with no differences at other time points. **Conclusions:** In this exploratory ancillary analysis of the NEOVASC trial, prolonging an exclusive EHMD until 36 weeks postmenstrual age was not associated with consistent differences in early brain maturation or motor performance. Given the high overall exposure to human milk in both groups, subtle effects may have been attenuated. These findings require confirmation in larger, adequately powered studies with long-term follow-up.

## 1. Introduction

Preterm birth, defined as birth before 37 completed weeks of gestation, remains a major global health challenge. In 2020, approximately 9.9% of all live births worldwide (13.4 million infants) were preterm [1]. In Europe, the prevalence of very preterm birth ranges from 1.1% to 1.6% [2]. In Austria, approximately 1000 of 77,000 live-born infants are born very preterm each year, including 60–80 infants in Tyrol, the region of the present study [3].

Prematurity represents a leading cause of neonatal mortality and morbidity, with the highest risk observed in infants born at very low gestational age (<32 weeks). Advances in perinatal and neonatal care over recent decades—including antenatal corticosteroid administration, improved respiratory support, and infection prevention—have substantially increased survival rates [4]. However, despite these improvements, preterm birth remains strongly associated with long-term neurodevelopmental impairment [5]. While the incidence of cerebral palsy has declined, rates of cognitive, behavioral, and socio-emotional difficulties remain high among children and young adults born preterm [5]. More than half of very preterm infants exhibit psychomotor or cognitive delays in early childhood, often persisting into later life, highlighting the need to identify modifiable factors that may improve long-term outcomes [6,7,8,9,10,11].

Early postnatal nutrition is increasingly recognized as a key modifiable determinant of brain development in preterm infants. Exposure to human milk during the neonatal period has been associated with improved brain maturation and more favorable neurodevelopmental outcomes [12,13,14,15,16,17]. Accordingly, the American Academy of Pediatrics recommends mothers’ own milk as the primary diet for very-low-birth-weight infants, with higher intake associated with greater benefit [17,18]. Because unfortified human milk does not fully meet the nutritional requirements of very preterm infants, fortification is recommended to support optimal growth [18,19]. Two main strategies are currently used: bovine milk-based fortifiers and human milk-based fortifiers, the latter enabling an exclusive human milk-based diet (EHMD). While human milk-based fortification is increasingly used in clinical practice, evidence regarding its impact on neurodevelopmental outcomes compared with bovine milk-based fortification remains limited [20,21]. Even less is known about the potential benefits of prolonging EHMD, including human milk-based fortification beyond the early postnatal period.

Therefore, the aim of this ancillary analysis of the NEOVASC trial was to investigate whether prolonging an EHMD until 36 weeks postmenstrual age, compared with the introduction of bovine milk-based products from 32 weeks, influences early brain development as assessed by magnetic resonance imaging at term-equivalent age and motor outcomes until 52 weeks postmenstrual age in very preterm infants.

## 2. Methods

### 2.1. Study Design and Participants

This study was conducted as an ancillary analysis of the NEOVASC randomized controlled trial (ClinicalTrials.gov Identifier: NCT04413994, date: 26 May 2020), a multicenter study performed in four Austrian tertiary neonatal centers [22]. The original NEOVASC trial was designed to evaluate the cardiovascular effects of a prolonged EHMD in preterm infants at preschool age. Details of the study protocol have been published previously [22]. This work represents an ancillary (secondary) analysis of data derived from the parent trial, addressing neurodevelopmental and neuroimaging outcomes in the first weeks of life not included among the original primary endpoints.

For the current analysis, only infants enrolled at the Medical University of Innsbruck were included. A total of 57 infants were initially recruited at this center. Three infants (one in the intervention group and two in the control group) died within the first days of life. The final study population, therefore, consisted of 54 preterm infants.

Eligible participants were preterm infants born before 32 completed weeks of gestation with a birth weight between 500 and 1250 g. Eligibility criteria for the NEOVASC trial were published previously [22]. In brief, exclusion criteria included birth weight < 500 g or >1250 g, inborn errors of metabolism (e.g., galactosemia or phenylketonuria), presence of major congenital malformations or chromosomal disorders, intestinal perforation or ≥stage II necrotizing enterocolitis prior to enrollment, absence of prior exclusive human milk feeding or nil per os status at enrollment, parental refusal of informed consent, and any condition deemed by the investigators to preclude participation (e.g., anticipated inability to survive the study period).

### 2.2. Nutritional Intervention

Infants were randomized in a 1:1 ratio to one of two feeding strategies:

1. **Intervention group:** EHMD consisting of mothers’ own milk or pasteurized donor human milk in combination with a human milk-based fortifier (Humavant™ + 6; Prolacta Bioscience, Inc., Duarte, CA, USA) administered continuously until 36 weeks postmenstrual age.
2. **Control group:** Until 32 weeks postmenstrual age, infants were fed according to our internal standards: Mothers’ own milk was prioritized, pasteurized donor human milk was used if maternal milk was unavailable, and preterm formula was given if no human milk was available. A human milk-based fortifier was administered as long as human milk was available. From 32 weeks onward, all infants in the control group received bovine milk, either as a bovine milk-based fortifier (if human milk was available) or as preterm formula.

This pragmatic design reflects routine clinical decision-making, which is primarily guided by birth weight in very preterm infants.

### 2.3. Patient Characteristics

Maternal, perinatal, and neonatal data were collected from electronic medical records.

Maternal variables included maternal age at delivery, diabetes, hypertensive disorders, and smoking during pregnancy (self-reported, yes/no).

Perinatal variables comprised antenatal corticosteroid exposure (any and complete course), antenatal magnesium sulfate administration, premature rupture of membranes (defined as rupture > 24 h before delivery), and mode of delivery.

Neonatal variables included sex, gestational age, birth weight, head circumference at birth, and clinical outcomes during hospitalization. An extremely preterm birth was defined as <28 weeks of gestation, and an extremely low birth weight as <1000 g. Small for gestational age (SGA) was defined as birth weight below the 10th percentile according to Fenton growth charts [23].

Early-onset sepsis was defined as infection occurring within the first 72 h of life, and late-onset sepsis as occurring thereafter, both requiring antibiotic treatment for at least five days. Respiratory support variables included surfactant administration and the need for mechanical ventilation. Cranial ultrasound was performed routinely to detect cerebral hemorrhage.

### 2.4. MRI Acquisition

MRI was performed at term-equivalent age using a 3.0 Tesla system without sedation during natural sleep. Image analyses were conducted by investigators blinded to group allocation.

### 2.5. Assessment of Brain Injury

Three-dimensional T1-weighted MP-RAGE and susceptibility-weighted sequences were used for the detection of brain injury. Intraventricular hemorrhage, white matter injury, and cerebellar hemorrhage were classified according to published criteria [24]. Depending on the severity, brain injury was classified as grade 1 to 4. When multiple grades were present, the higher grade was assigned. High-grade injury (grades 3–4) in any category was classified as severe brain injury.

Intraventricular hemorrhage grades were defined as follows: grade 1, hemosiderin deposits or post-hemorrhagic cysts within the thalamo-caudal notches; grade 2, hemosiderin deposits along the ventricular wall without ventricular dilatation; grade 3, ventricular dilatation above the 97th percentile with evidence of previous hemorrhage; and grade 4, parenchymal hemorrhage or post-hemorrhagic cystic encephalomalacia.

White matter injury was graded as follows: grade 1–2, punctate lesions ≤ 3 mm in the periventricular white matter; grade 2 was distinguished from grade 1 by the presence of lesions in bilateral corticospinal tracts or, more extensively, with >3 lesions per hemisphere; grade 3, extensive lesions along the ventricular wall with T1 hyperintensity; and grade 4, cystic lesions in the periventricular white matter.

Cerebellar hemorrhage was graded as follows: grade 1, unilateral punctate lesions ≤ 3 mm; grade 2, bilateral punctate lesions; grade 3, unilateral lesion > 3 mm; and grade 4, extensive bilateral lesions.

Images were reviewed in interdisciplinary consensus meetings (neonatology, neuroradiology, and neuropediatrics).

### 2.6. Assessment of Brain Growth

Established two-dimensional MRI metrics were applied to assess regional brain size and extracerebral fluid spaces. On coronal T1-weighted images, biparietal width (BPW), interhemispheric distance (IHD), parietal extracerebral space (parietal ECS), and transcerebellar diameter (TCD) were measured according to previously published protocols [25,26,27]. Temporal lobe length (TLL) and temporal extracerebral space (temporal ECS) were assessed on sagittal T1-weighted images. TLL was determined on three consecutive slices, and the longest measurement per hemisphere was used for analysis. Temporal ECS was measured on the same slice as the linear continuation of the TLL [28]. For bilateral parameters (parietal and temporal ECS and TLL), mean values of both hemispheres were used for further analysis. Interrater reliability for all metrics was high (intraclass correlation coefficient [ICC] > 0.90), and intra-observer agreement was excellent (ICC > 0.93), as previously reported [26,27]. For the present study, all measurements were performed by a single investigator.

Cerebral microstructure was assessed using axial diffusion-weighted imaging covering the whole brain (matrix 160 × 160; b = 1000 s/mm^2^; 20 diffusion directions repeated twice; TE/TR 101/6600 ms). Fractional anisotropy (FA) and apparent diffusion coefficient (ADC) were measured in predefined regions of interest, including the centrum semiovale (right/left), posterior limb of the internal capsule (right/left), the genu and splenium of the corpus callosum, occipital white matter (right/left), cerebellum (right/left), and the middle cerebellar peduncles (right/left), as previously described [29]. Regions of interest were manually placed on FA maps and transferred to ADC maps.

Inter- and intra-rater reliability were previously shown to be good to excellent (intraclass correlation coefficient 0.68–0.98) [29]. For the present analysis, measurements performed by a single investigator were used.

### 2.7. Assessment of General Movement Optimality Scores (GMOS) and Motor Optimality Score (MOS)

The General Movement Assessment, according to the optimality concept, allows for a quantitative evaluation of movement quality and repertoire and is a validated tool for early detection of neuromotor impairment [30].

Videos for the assessment of general movements (GMs) were recorded at four predefined time points: preterm age (at 34 and 36 weeks postmenstrual age), term-equivalent age (at 40 weeks postmenstrual age), and post-term age (at 52 weeks postmenstrual age). Each infant was filmed in a supine position, dressed in minimal clothing, and in an active behavioral state, avoiding episodes of crying or fussing, according to the Prechtl methodology [31]. For each session, recordings of about 3 min containing at least three spontaneous GM sequences were obtained.

Assessment of GMs at 34, 36, and 40 weeks postmenstrual age was performed using the GMOS, applying Prechtl’s optimality concept [32]. The GMOS rates the quality of movement components—including sequence, amplitude, speed, spatial range, rotations, onset and offset, tremulousness, and stiffness—for neck, trunk, and upper and lower extremities. Each item is scored as optimal (2), suboptimal (1), or non-optimal (0), yielding a total score ranging from 5 to 42, with higher values indicating better motor performance.

Assessment of motor performance at 52 weeks postmenstrual age was evaluated using the MOS comprising five domains [33]: (1) fidgety movements, (2) age-adequate motor repertoire, (3) quality of movement patterns, (4) posture, and (5) overall quality of movement motor repertoire, including fluency, smoothness, and variability. Each domain is scored individually, resulting in a total score ranging from 5 (most abnormal) to 28 (optimal), with higher scores reflecting better motor development.

Videos were analyzed in weekly interdisciplinary meetings by certified observers trained at the GM Trust (basic and advanced levels), who were blinded to group allocation and clinical information.

### 2.8. Statistical Analysis

Statistical analyses were performed with IBM SPSS Statistics Version 29.0 for Windows. The distribution of continuous variables was assessed using the Shapiro–Wilk test. Continuous variables are reported as median with interquartile range (IQR) and were analyzed using the Mann–Whitney U test. Categorical variables are presented as absolute numbers and percentages and were compared using the chi-square test or Fisher’s exact test.

Because maternal smoking during pregnancy differed between groups and represents a plausible confounder, adjusted linear regression analyses were performed for outcomes showing significant unadjusted between-group differences. In addition, a non-significant trend toward a higher proportion of small-for-gestational-age infants in the intervention group was observed; given its potential relevance as a confounder, this variable was also included in the adjusted models.

Missing data are indicated in the respective table footnotes. A two-sided *p* value < 0.05 was considered statistically significant.

### 2.9. Ethical Approval

The study was approved by the Ethics Committee of the Medical University of Innsbruck (Study No. AN2013-0086 and 1317/2019, approval date 2 March 2020) and was conducted in accordance with the Declaration of Helsinki and its later amendments. Written informed consent to participate was obtained from the parents or legal guardians of all participants prior to study enrollment.

### 2.10. Use of AI Tools

A large language model (ChatGPT, OpenAI, San Francisco, CA, USA, **based on the GPT-5 architecture**) was used to assist with language editing and improvement of wording. All generated content was critically reviewed, verified, and approved by the authors, who take full responsibility for the accuracy and originality of the manuscript.

## 3. Results

### 3.1. Maternal and Neonatal Characteristics

A total of 54 preterm infants (53.7% female) with a median gestational age of 28.6 weeks (IQR 26.7–29.6) were included. Among maternal variables, smoking during pregnancy differed significantly between groups, occurring in none of the infants in the intervention group and in 4/26 (15.4%) in the control group (*p* = 0.031). No other maternal characteristics differed significantly.

Perinatal variables were comparable between groups. Among neonatal characteristics, there was a non-significant trend toward a higher proportion of small-for-gestational-age infants in the intervention group (*p* = 0.069). No further differences were observed in other neonatal variables. Detailed data are presented in Table 1.

### 3.2. Brain Injury and Brain Growth

No differences were observed between groups in the prevalence or severity of white matter injury, cerebellar hemorrhage, or intraventricular hemorrhage. Given the small number of affected infants, IVH laterality was not subjected to formal statistical analysis but is presented descriptively; within this descriptive overview, a higher number of severe right-sided IVH cases was observed in the intervention group. Detailed information on lesion distribution is provided in the Supplementary Table S1. Quantitative brain growth metrics also did not differ between groups. Detailed results are shown in Table 2.

### 3.3. Microstructural Brain Maturation

Unadjusted analyses showed a lower median FA in the right posterior limb of the internal capsule in the intervention group (0.59 [0.56–0.61] vs. 0.62 [0.60–0.64]; *p* = 0.019). Additionally, median ADC in the left middle cerebellar peduncle was lower in the intervention group (1.00 [0.97–1.07] vs. 1.05 [1.00–1.08]; *p* = 0.027). No other significant differences in diffusion tensor imaging metrics were observed. Detailed results of the unadjusted analyses are presented in Supplementary Figures S1 and S2.

After adjustment for small-for-gestational-age status, the difference in FA in the right posterior limb of the internal capsule was no longer statistically significant (adjusted R^2^ 0.106; F[2,48] = 3.979; *p* = 0.101). Similarly, after adjustment for maternal smoking during pregnancy, the difference in ADC in the left middle cerebellar peduncle was no longer statistically significant (adjusted R^2^ < 0; F[2,46] = 0.478; *p* = 0.623).

### 3.4. General Movement Optimality Scores (GMOS) and Motor Optimality Score (MOS)

GMOS were available for 51/57 (89.5%) infants at 34 weeks, 53/57 (93.0%) at 36 weeks, and 51/57 (89.5%) at 40 weeks postmenstrual age. MOS at 52 weeks postmenstrual age was available for 52/57 (91.2%) infants.

Median GMOS at 40 weeks postmenstrual age was higher in the intervention group compared with controls (33.0 [28.0–39.0] vs. 28.5 [25.6–31.0]; *p* = 0.011). No significant differences were observed at the other assessed time points. Detailed GMOS results are presented in Table 3.

Given the imbalance in maternal smoking during pregnancy between groups, an adjusted linear regression analysis was performed for GMOS at 40 weeks postmenstrual age. The between-group difference remained statistically significant after adjustment (adjusted R^2^ = 0.092; F[2,48] = 3.521; *p* = 0.037).

## 4. Discussion

Previous studies have suggested that early exposure to human milk supports white matter maturation and neurodevelopmental outcomes [12,13,14,15,34]. However, evidence for the additional benefit of prolonging EHMD is limited. In this ancillary study of the NEOVASC randomized controlled trial, preterm infants receiving a prolonged EHMD until 36 weeks postmenstrual age did not show consistent differences in (micro)structural brain maturation or early motor development compared with infants receiving a diet including bovine milk-based components from 32 weeks postmenstrual age.

Overall, quantitative MRI-based measures of brain structure and diffusion metrics were largely comparable between groups. A single isolated finding of FA in the right posterior limb of the internal capsule in the intervention group was observed in unadjusted analyses. However, this difference was no longer statistically significant after adjustment for small-for-gestational-age status, suggesting that it may be driven by baseline differences in growth status rather than a true nutritional effect. In addition, a higher proportion of severe right-sided intraventricular hemorrhage in the intervention group may have contributed to this observation. Given the absence of consistent differences across other white matter regions, this finding should be interpreted cautiously and considered hypothesis-generating and requires confirmation in future studies. In contrast to our findings, two previous neuroimaging studies reported beneficial effects of early human milk exposure on cortical maturation and brain connectivity [12,13]. However, these findings are based on varying degrees of human milk exposure (e.g., ≥75% vs. <75% of inpatient days), rather than clearly defined feeding strategies. Differences in the quantity and duration of human milk intake likely contribute to heterogeneity in reported outcomes. Human milk-based vs. bovine milk-based fortification was not systematically evaluated in these studies, further limiting direct comparability with the present randomized trial.

Functional assessment using the GMOS revealed higher median scores at 40 weeks postmenstrual age in the intervention group, suggesting slightly more advanced neuromotor performance at this specific time point. However, no differences were observed at other assessed ages, indicating that any effect may be limited to the duration of the intervention and, in the absence of differences at 52 weeks, is unlikely to represent a clinically meaningful finding.

Several factors may explain the limited differences observed in this study.

First, a contributing major factor may be the relatively high exposure to human milk in both groups. In line with current recommendations and our institutional feeding standards, human milk is prioritized whenever possible during the early postnatal period [18]. Consequently, only 42% of infants in the control group were exposed to bovine milk before 32 weeks postmenstrual age (preliminary data). As a result, the nutritional contrast between groups primarily reflects the duration of exclusive human milk-based fortification, rather than substantial differences in the initial type of enteral nutrition. This overlap may have reduced the ability to detect subtle intervention effects.

Current evidence supports a clear hierarchy for feeding very preterm infants with respect to neurodevelopmental outcomes, with mothers’ own milk associated with the most favorable outcomes, followed by donor human milk and then formula feeding [14,15,17]. A dose–response relationship has been consistently demonstrated for mothers’ milk, with increasing exposure associated with improvements in cognitive, language, and motor outcomes that may persist into school age and beyond [14,15,17].

While the benefits of mothers’ own milk are well established, evidence regarding the neurodevelopmental effects of different fortifier types, specifically human milk-based versus bovine milk-based fortifiers, remains limited and inconsistent [21]. In studies comparing these fortifiers, infants are exposed to heterogeneous feeding regimens, including combinations of mother’s milk, donor human milk, and preterm formula as the base diet. In addition, variations in fortification strategies further complicate direct comparisons across trials. These differences in both the quantity and quality of human milk exposure substantially limit the comparability of findings across studies.

While Sullivan et al. reported a reduced incidence of NEC in infants receiving an exclusive human milk-based diet including a human milk-derived fortifier compared to a mixed feeding regimen including mother’s milk + formula + bovine milk-derived fortifier, and a large multicenter randomized controlled trial from Sweden found no benefit of human milk-derived over bovine milk-derived fortifier in preventing NEC, sepsis, or mortality in extremely preterm infants fed an exclusive human milk diet [35,36]. Neurodevelopmental outcomes in the latter cohort have not yet been reported [36]. A 2019 Cochrane review identified only one randomized controlled trial comparing human milk-derived fortifier with bovine milk-derived fortifier in preterm infants fed exclusively with human milk [21,37]. A follow-up analysis of this cohort reported no significant differences in Bayley-III cognitive, language, or motor composite scores at 18 months corrected age [20].

Second, the nutritional divergence between groups began relatively late (from 32 weeks postmenstrual age). Consequently, the window for differential neurodevelopmental effects of prolonged EHMD before the first assessment was limited, and any effects may have been difficult to detect given the sample size.

Third, beyond providing essential nutrients and bioactive factors that support neurodevelopment, human milk feeding may also serve as a marker of maternal care, being associated with increased emotional interaction, maternal sensitivity, stronger infant attachment, and greater mother–infant engagement during feeding [38,39,40]. Because the intervention consisted solely of prolonged fortification with a human milk-based fortifier, the non-nutritional benefits of human milk were likely similar in both groups, further explaining the limited additional effect of the intervention.

A number of limitations should be considered when interpreting the present findings.

This ancillary analysis was not powered to detect differences in the neuroimaging or neurodevelopmental outcomes examined; therefore, the study is exploratory in nature, and all findings should be considered hypothesis-generating. As such, isolated statistically significant findings may reflect chance rather than true biological effects. In addition, MRI and general movement assessments were performed at a single center, which may limit generalizability. Furthermore, as brain maturation was assessed at an early time point, potential group differences may not yet have been fully apparent and could emerge only at later developmental stages.

Despite randomization, some imbalance in baseline characteristics may have influenced the results. Moreover, maternal and perinatal factors beyond those captured in the dataset, including non-nutritional aspects of human milk feeding such as maternal–infant interaction, were not assessed and may represent potential sources of residual confounding.

Both study groups had a high overall exposure to human milk in the early postnatal period, which is in line with institutional feeding standards. As a result, the nutritional contrast between groups was primarily driven by differences in the duration of exclusive human milk-based fortification rather than major differences in initial feeding type, which may have reduced the ability to detect subtle intervention effects.

Strengths of this study include the randomized, prospective design, the well-characterized cohort of preterm infants, and the integration of structural (MRI/DTI) and functional (GMOS) assessments. All MRI and DTI acquisitions were standardized, and analyses were performed by experienced investigators blinded to group allocation, minimizing measurement bias and strengthening internal validity.

## 5. Conclusions

In this ancillary analysis of the NEOVASC randomized controlled trial, prolonging an exclusive human milk-based diet until 36 weeks postmenstrual age was not associated with consistent differences in early brain structural maturation or functional motor outcomes compared with earlier introduction of bovine milk-based products in very preterm infants.

Given the exploratory nature of the analysis, the limited sample size, and the high overall exposure to human milk in both groups, these findings should be interpreted with caution and considered hypothesis-generating rather than confirmatory. Residual confounding and limited statistical power cannot be excluded.

Larger, adequately powered studies with detailed nutritional exposure data and long-term follow-up are required to determine whether prolonged exclusive human milk-based feeding has clinically meaningful effects on neurodevelopmental outcomes in very preterm infants. Consistent with this, ongoing follow-up of the multicenter NEOVASC cohort is expected to provide important insights into neurodevelopmental outcomes at preschool age.

## Figures and Tables

**Table 1:** Maternal and neonatal characteristics in the intervention and control groups.

	Intervention Group (*n* = 28)	Control Group (*n* = 26)	*p*-Value
	*n* (%) or Median (IQR)		
**Maternal data**			
Maternal age at pregnancy [years]	32.0 (29.0; 36.5)	34.0 (29.0; 37.3)	0.381
Maternal diabetes	3 (10.7)	2 (7.7)	0.788
Maternal hypertensive disorder	7 (25.0)	5 (19.2)	0.610
Maternal smoking during pregnancy	0	4 (15.4)	**0.031**
**Perinatal data**			
Antenatal administration of any steroids	27 (96.4)	24 (92.3)	0.509
Antenatal administration of a full course of steroids	22 (78.6)	16 (61.5)	0.171
Antenatal administration of magnesium sulfate	18 (64.3)	22 (84.6)	0.089
Premature rupture of membranes	6 (21.4)	4 (15.4)	0.568
Delivery by cesarean section	28 (100)	26 (100)	1.000
**Neonatal data**			
Sex, male/female	10 (35.7)/18 (64.3)	15 (57.7)/11 (42.3)	0.106
Gestational age [weeks]	28.9 (26.7; 29.5)	28 (26.6; 29.6)	0.585
Extremely preterm infants	11 (39.3)	12 (46.2)	0.435
Birth weight [g]	1000 (855; 1118)	1025 (943; 1181)	0.377
Extremely low birth weight	14 (50.0)	10 (38.5)	0.554
Small for gestational age (SGA)	9 (32.1)	3 (11.5)	0.069
Head circumference at birth [cm]	26.0 (24.5; 26.9)	26.5 (25.5; 27.0)	0.426
Administration of surfactant	24 (85.7)	24 (92.3)	0.267
Need for mechanical ventilation	14 (50.0)	14 (53.8)	0.586
Need for supplemental oxygen at 36 weeks of gestation	3 (10.7)	4 (15.4)	0.610
Early-/late-onset sepsis	8 (28.6)	4 (15.4)	0.244
Time to full enteral feeding [days]	11 (10; 16)	13 (11; 17)	0.098
Administration of any postnatal steroids	5 (17.9)	5 (19.2)	0.626
Any cerebral hemorrhage on cerebral ultrasound	5 (17.9)	5 (19.2)	0.897
Postmenstrual age at discharge [weeks]	38.1 (36.9; 39.5)	38.1 (36.4; 39.0)	0.579

**Table 2:** Brain injury and quantitative MRI brain growth metrics in the intervention and control groups.

	Intervention Group (*n* = 28)	Control Group (*n* = 26)	*p*-Value
	*n* (%) or Median (IQR)		
Brain injury			
Any injury on MRI	10 (35.7)	6 (23.1)	0.340
White matter injury	5 (17.9)	3 (11.5)	0.518
White matter injury grade 3–4	0 (0.0)	0 (0.0)	1.000
Cerebellar hemorrhage	1 (3.6)	1 (3.8)	0.958
Cerebellar hemorrhage grade 3–4	0 (0.0)	0 (0.0)	1.000
Intraventricular hemorrhage	8 (28.6)	4 (15.4)	0.249
Intraventricular hemorrhage grade 3–4	3 (10.7)	1 (3.8)	0.340
Quantitative Brain Growth Metrics			
Biparietal width	75.5 (74.0; 78.5)	76.9 (75.4; 79.5)	0.105
Interhemispheric distance	2.9 (2.0; 3.8)	3.0 (2.3; 4.9)	0.305
Parietal extracerebral space	2.8 (2.1; 4.4)	3.2 (2.7; 4.1)	0.446
Transcerebellar diameter	52.5 (50.7; 54.5)	53.4 (51.9; 55.3)	0.139
Temporal lobe length	84.8 (82.2; 87)	83.7 (81.4; 86.8)	0.522
Temporal extracerebral space	2.7 (1.9; 3.2)	3.2 (2.1; 4.1)	0.327

**Table 3:** General Movement Optimality Scores (GMOS) at 34, 36, and 40 weeks postmenstrual age, and Motor Optimality Score (MOS) at 52 weeks postmenstrual age in the intervention and control groups.

	Intervention Group (*n* = 28)	Control Group (*n* = 26)	*p*-Value
	*n* (%) or Median (IQR)		
GMOS 34 postmenstrual weeks	28.5 (23.3; 36.0)	26.0 (22.0; 31.0)	0.271
GMOS 36 postmenstrual weeks	33.0 (25.0; 39.8)	28.0 (25.0; 33.5)	0.059
GMOS 40 postmenstrual weeks	33.0 (28.0; 39.0)	28.5 (25.8; 31.0)	**0.011**
MOS 52 postmenstrual weeks	26.0 (24.0; 26.0)	26.0 (23.0; 28.0)	0.732

Bold values indicate statistical significance (*p* < 0.05).

## Data Availability

All data generated or analyzed during this study are included in this article. Further enquiries can be directed to the corresponding author.

## Supplementary Materials

The following supporting information can be downloaded at: https://www.mdpi.com/article/10.3390/nu18091321/s1; Supplementary Table S1: Distribution of intraventricular hemorrhage (IVH) according to laterality and severity. Supplementary Figure S1: Boxplots of fractional anisotropy (FA) values across all predefined regions of interest in the intervention and control groups. Supplementary Figure S2: Boxplots of apparent diffusion coefficient (ADC) values across all predefined regions of interest in the intervention and control groups.

## Abbreviations

ADC	Apparent diffusion coefficient
BPW	Biparietal width
CB	Cerebellum
CCg	Corpus callosum genu
CCs	Corpus callosum splenium
DTI	Diffusion tensor imaging
ECS	Extracerebral space
EHMD	Exclusive human milk-based diet
FA	Fractional anisotropy
GM	General movements
GMOS	General Movement Optimality Score
IHD	Interhemispheric distance
IQR	Interquartile range
IVH	Intraventricular hemorrhage
MCP	Middle cerebellar peduncle
MRI	Magnetic resonance imaging
MOS	Motor Optimality Score
OcWM	Occipital white matter
PLIC	Posterior limb of internal capsule
SGA	Small for gestational age
TCD	Transcerebellar diameter
TLL	Temporal lobe length
