# Supplementary material for: Effects of a Prolonged Exclusive Human Milk-Based Diet on Structural and Functional Brain Maturation in Very Preterm Infants: An Ancillary Analysis of the NEOVASC Trial
Source: Nutrients. 2026 Apr 22;18(9):1321. doi: 10.3390/nu18091321 (PMC13164981; doi:10.3390/nu18091321)
Supplement: Supplementary file 1 [file nutrients-18-01321-s001.zip › Supplementary Table S1.pdf]

Supplementary Table S1: Distribution of intraventricular hemorrhage (IVH) according to laterality and severity.

	IVH	Right	Left
**Intervention group**			
	Grade 1		x
	Grade 1	x	
	Grade 2	x	
	Grade 2	x	x
	Grade 2	x	x
	Grade 3	x	x
	Grade 4	x	
	Grade 4	x	
**Control group**			
	Grade 2	x	
	Grade 2	x	x
	Grade 2	x	x
	Grade 3		x
