# Supplementary material for: Effects of a Prolonged Exclusive Human Milk-Based Diet on Structural and Functional Brain Maturation in Very Preterm Infants: An Ancillary Analysis of the NEOVASC Trial
Source: Nutrients. 2026 Apr 22;18(9):1321. doi: 10.3390/nu18091321 (PMC13164981; doi:10.3390/nu18091321)
Supplement: Supplementary file 1 [file nutrients-18-01321-s001.zip › Supplementary Figure 1.pdf]

**Supplementary Figure 1: Boxplots of fractional anisotropy (FA) values across all predefined regions of interest in the intervention and control groups.**

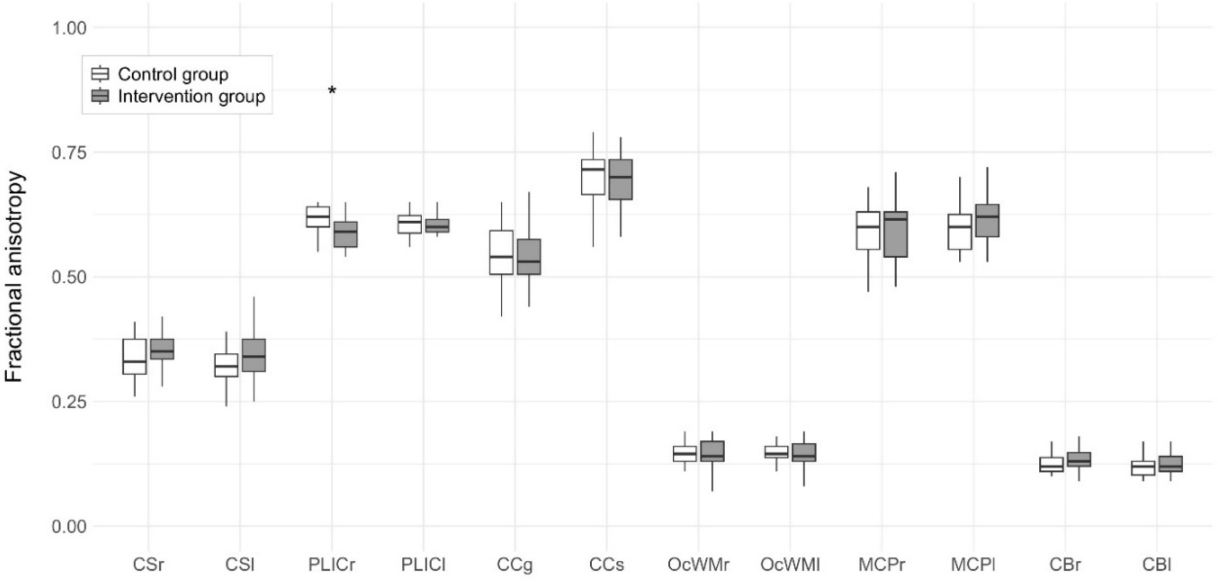


CS, centrum semiovale; PLIC, posterior limb of internal capsule; CCg, corpus callosum genu; CCs, corpus callosum splenium; OcWM, occipital white matter; CB, cerebellum; MCP, middle cerebellar peduncle; r, right; l, left. *$p < 0.05$.
